# Evaluating the effectiveness of lateral postural management for breech presentation: study protocol for a randomized controlled trial (BRLT study)

**DOI:** 10.1186/s13063-023-07395-w

**Published:** 2023-05-27

**Authors:** Hiroki Shinmura, Takashi Matsushima, Asako Watanabe, Honglian Shi, Asako Nagashima, Ayako Takizawa, Mayu Yamada, Eika Harigane, Youhei Tsunoda, Ryuhei Kurashina, Go Ichikawa, Shunji Suzuki

**Affiliations:** Department of Obstetrics and Gynecology, Nippon Medical School Musashikosugi Hospital, 1-383 Kosugicho, Nakahara-Ku, Kawasaki-Shi, Kanagawa, 211-8533 Japan

**Keywords:** Maternal medicine, Ultrasonography, Prenatal diagnosis, Sleep medicine, Randomized controlled trial

## Abstract

**Background:**

Breech presentation is observed in 3–4% at term of pregnancy and is one of the leading causes of cesarean section. There is no established treatment for breech presentation before 36 weeks. A retrospective cohort study was conducted to demonstrate that the lateral position is effective for breech presentation. However, there are no randomized controlled trials evaluating lateral position management for breech presentation. Here, we described the methodology of a randomized controlled trial of a cephalic version for breech presentation in the third trimester by lateral postural management (BRLT study).

**Methods:**

The BRLT study is an open-label, randomized controlled trial with two parallel groups allocated in a 1:1 ratio to examine the lateral position management for breech presentation, as compared with expectant management care. An academic hospital in Japan will enroll 200 patients diagnosed with a breech presentation by ultrasonography between 28 + 0 weeks and 30 + 0 weeks. Participants in the intervention group will be instructed to lie on their right sides for 15 min three times per day if the fetal back was on the left side or lie on their left sides if the fetal back was on the right side. The instruction will be given every 2 weeks after confirmation of fetal position, and the lateral position will be instructed until the cephalic version, and after the cephalic version, the reverse lateral position will be instructed until delivery. The primary outcome is cephalic presentation at term. The secondary outcomes are cesarean delivery, cephalic presentation 2, 4, and 6 weeks after the instruction, and at delivery, recurrent breech presentation after cephalic version, and adverse effects.

**Discussion:**

This trial will answer whether the lateral positioning technique is effective in treating breech presentation and, depending on the results, may provide a very simple, less painful, and safe option for treating breech presentation before 36 weeks, and it may impact breech presentation treatment.

**Trial registration:**

UMIN Clinical Trials Registry UMIN000043613. Registered on 15 March 2021 https://center6.umin.ac.jp/cgi-open-bin/ctr_e/ctr_view.cgi?recptno=R000049800.

**Supplementary Information:**

The online version contains supplementary material available at 10.1186/s13063-023-07395-w.

## Background

Breech presentation is a common abnormal presentation that occurs in 3–4% at term [1]. It is a leading cause of cesarean section because of increased perinatal morbidity and mortality [2, 3]. Thus, the cephalic version is a clinically important topic since it can minimize the need for unnecessary cesarean sections. Presently, the recommended method for breech presentation is the external cephalic version performed at 36 to 37 weeks [4–7]. However, it requires a well-equipped facility due to the risk of fetal distress, premature membrane rupture, placental abruption, and abdominal pain [8–10]. In contrast, there is a paucity of evidence on treatments for the breech presentation that can be applied before 36 weeks, and no recommended methods exist in developed countries [11–17]. According to a Cochrane review, there was insufficient evidence to support the use of postural therapy for breech presentation [12]. The effectiveness of acupuncture and moxibustion therapy for breech presentation is also controversial [13–17].

Since Taoka reported the lateral position for breech presentation in 1943, it has been practiced along with the knee–chest position in Japan for a long time [18]. The lateral position is thought to promote fetus self-rotation using the effect of gravity. In a prospective cohort study using MRI data, in late gestation pregnancy, the lateral position was considered safer than the supine position, increasing internal iliac artery blood flow by 23.7% [19]. A meta-analysis reported that it was safe to sleep in the lateral position on the right or left sides during pregnancy [20]. The lateral position management for breech presentation is very simple, safe, less painful, cost free, and does not require complex instruction, so it can be easily implemented worldwide. Japanese obstetricians have realized the effectiveness of the lateral position alone, but perhaps because it is so simple, its specific effects have not been validated by studies with sufficient evidence [21]. In the Cochrane review described above, four of the six randomized well-controlled trials on postural therapies for the breech presentation were on the knee–chest position, two on the Indian version, and no studies on the lateral position were included [12].

In a previous retrospective cohort study, our group discovered the lateral position alone was effective for primiparous breech presentation between 28 + 0 and 29 + 6 weeks [22]. In the study, a single instruction of the lateral position significantly reduced primiparous breech fetuses 2 weeks after the instruction, but no statistically significant difference in the cesarean section rate, although a difference was observed in the data [22]. Since it was thought that the effect size was small in a single session of instruction, multiple serial sessions of instruction were planned to increase the effect size. It may have a cumulative effect on the breech presentation. Based on the lack of adverse effects in the previous study, leading to the conclusion that serial instruction of the lateral position, which is expected to be more effective, could be safely implemented. According to the original lateral position method of Taoka, the position of the fetal back defines which side to be taught, but in the previous study, 38% of the fetuses were in the opposite side at the next check-up 2 weeks later [22]. In addition, sleep onset position is the dominant position overnight for half of at a median gestation of 34 weeks [23]. Hence, we thought it is necessary to update every 2 weeks the left and right orientation to be taught. Furthermore, if the patient was in the cephalic position, it was necessary to teach the opposite side, so we thought it was necessary to adjust the direction of instruction at each visit. Based on the above evidence, it was considered that it would be practically possible to set up a new trial of lateral position with a higher level of evidence to prove the effectiveness of the lateral positioning method for breech presentation.

This is the first randomized controlled clinical trial to evaluate the effect of the lateral position instruction on breech presentation. Simultaneously, we established a study to evaluate the effectiveness of serial instruction in reducing cesarean sections for breech presentation. The serial instruction was planned with a combination of lateral positions and the reverse lateral positions, which is theoretically thought to prevent reverting to breech presentation after the cephalic version. Since the lateral position method is thought to promote fetal self-rotation, it is possible that a higher volume of amniotic fluid may facilitate rotation. Therefore, it was thought that the effect of continuous instruction would be maximized if instruction began by 32 weeks, when amniotic fluid volume is reported to be at its peak [24], so 28–30 weeks was chosen for inclusion in the study.

The primary objective of this trial is to determine whether serial instructions of the lateral or reverse lateral positions can minimize the number of fetuses in breech presentation at term.

## Methods

### Trial design and setting

This trial is designed as an open-label, randomized controlled trial with two parallel groups allocated in a 1:1 ratio to evaluate the superiority of lateral position management for breech presentation, as compared with expectant management care, at an academic hospital in Kawasaki (Kanagawa, Japan).

The protocol was designed in line with SPIRIT (Standard Protocol Items: Recommendations for Interventional Trials) 2013 (Additional file 1).

### Sample size and stratification

The sample size was calculated based on our previous retrospective cohort study and other prior studies. In our previous study, a rate of fetal cephalic version at term was 94% (16/17) in the lateral position group and 77% (30/39) in the control group without any intervention at all [22]. Hence, in this study, the expected percentage of cephalic fetuses at term in intervention group of lateral position was set at 94%. Typically, 20–25% of fetuses are in pelvic position at less than 28 weeks, dropping to 3–4% at term, with a cephalic conversion rate of 84% [25, 26]. In addition, Fox and Chapman reported in a study of 1010 patients that fetuses in pelvic position at 28–30 weeks, which is the number of weeks we have covered in our research, had a 75% chance of returning to the cephalic position [27]. Based on the above evidence, we set the predicted cephalic conversion rate at term in the control group at 80%. The effect size was calculated as *φ* = 0.2081454 using G*Power (version 3.1, Faul, Erdfelder, Lang, and Buchner, Düsseldorf, Germany) [28]. We set *α* = 0.05 (two-sided) and 1-β = 0.80, used *χ*2 test and required 182 participants. Considering the missing value to be approximately 10%, we selected 100 participants per arm, making a total of 200 participants. The secondary endpoints were not considered in the sample size calculation.

It was suggested that previous childbirth history had a significant effect on the results in postural management study for breech presentation [11], so we will stratify randomization based on parity (primipara or multipara).

### Inclusion criteria

The inclusion criteria are as follows:

1. Breech presentation diagnosed by ultrasonography
2. Gestational age between 28 + 0 and 30 + 0 weeks
3. Pregnant women undergoing prenatal check-ups at the Nippon Medical School Musashikosugi hospital

### Exclusion criteria

The exclusion criteria are as follows:

1. Less than 20 years old (we determined that there was a high possibility of deviating from the protocol for social reasons, since the consent of a parent or guardian is required for persons under 20 years of age to participate in clinical trials in Japan, and consent cannot be obtained if the parent or guardian is not present on the day of the enrollment.)
2. Scheduled cesarean delivery (included placenta previa, history of cesarean delivery, and history of myomectomy)
3. Scheduled delivery at another hospital
4. Multiple pregnancies
5. Transverse position
6. Any treatment already in place for breech presentation
7. Complications with difficulty in postural management

### Participant enrollment

Recruitment will be taken place at a single academic hospital. Trial notices will be published on the academic hospital web page, but no advertising will be used. To prevent recruitment omissions, eligibility will be evaluated when diagnosed with breech presentation, and all patients screened for research eligibility will be recruited on the same day, on the spot by the attending obstetricians. To prevent variation among recruiters, the explanation of the study is presented according to a pre-prepared document. Before randomization, written informed consent will be obtained from the patient if she agrees to participate. The informed consent documents are available upon reasonable request from the corresponding author.

### Randomization and allocation concealment

An independent researcher made the randomization list using the random function of Microsoft Excel 2019 (version 2111, Microsoft Corp., Redmond, WA, USA). Random block sizes were used, and the participants were stratified by parity (primiparous or multiparous). Because of the stratification by parity, two allocation lists were prepared, one for primiparas and the other for multiparas. The computer generated two consecutively numbered allocation lists which were printed and sealed with security void stickers. If the sticker is removed, the word “Void” will remain on the list. Moreover, additional sealing stickers were placed on the back of the list to prevent it from being seen through the allocation. A nurse from the outpatient section of obstetrics, who was not involved in the enrollment process, performed randomization by removing the stickers from the list in order. Recruiters and participants will not have access to the list. There will be no masking after randomization, and both the investigator and participant will know the group she has been assigned to. Trained outcome assessor and data analyst will not be told about group allocation.

### Interventions

Lateral position in this study is defined as taking the lateral recumbent position (also known as the lateral decubitus position) with the left or right side down, which side to lie on is determined by whether the fetus’ back is on the left or right side of the maternal body. Women assigned to the intervention group will be instructed to lie on their right side for 15 min three times per day for 2 weeks when the fetal back was on the left side or to lie on their left side when the fetal back was on the right side (lateral position). If the subject is in good physical condition, consecutive sessions of the lateral position will be permitted. In addition, if the patient could maintain the designated position for at least 15 min at the beginning of sleep, changing to another position, such as turning over, will be permitted. Antenatal check-up doctors who have undergone training in advance to avoid incorrect guidance will be in charge of providing guidance. To make it easier for participants to understand what position to take, show them a picture of a pregnant woman in the lateral position and provide them with simple written instructions which side to lie. Two weeks after the instruction, the participant will return to the hospital for the next prenatal check-up, and the position of the fetus will be confirmed by ultrasound again. If the fetus remains in the breech presentation, the attending obstetricians will repeat the instruction of lateral position based on the fetal back position. After the cephalic version, participants of the intervention group will be instructed on the other side. Specifically, in the case of cephalic presentation, the right lateral recumbent position is taken if the fetal back is on the right side of the maternal body, and the left lateral recumbent position is taken if the fetal back is on the left side of the maternal body (reverse lateral position). Pregnancy check-ups will be done every 2 weeks and guidance of lateral position or reverse lateral position will be given each time until delivery. If the fetus is placed in the transverse presentation during the study, we will instruct participants to lie on the left side when the fetal head is on the right side or to lie on the right side when the fetal head is on the left side. The knee–chest position and other management for breech presentation will not be recommended. Participants will be instructed to discontinue lateral position if they felt uncomfortable during the designated posture and report the incident. We will ask the participants to record a daily position record form to confirm whether they are correctly lying on their sides or not. From the collected daily record sheets, the implementation rate of the taught positions was calculated, and 50% or more was defined as successful implementation. The management of pregnancy other than the interventions will follow guidelines for obstetrical practice in Japan 2020.

Women of the control group will be received expectant management care complying with the guidelines for obstetrical practice in Japan 2020. Any other management for breech presentation will not be recommended. To reassure patients and prevent them from dropping out of the study, we will explain that postural, acupuncture, and moxibustion therapy are not recommended due to lack of evidence. In the control group, taking the lateral position will not be prohibited, although the proper side will not be taught deliberately.

All participants of the two groups will be asked to fill out a daily position record form until delivery to confirm the type of position they take. If the breech presentation is still present at term, all participants will be informed about external cephalic version and vaginal delivery or elective cesarean section. An elective cesarean section will be scheduled at 38 weeks. If participants hope, they will be referred to another hospital that provides those treatments. Participants will be allowed to withdraw from the study at any time for any reason and informed of that in advance. We will work to obtain permission to use the patient’s medical information for subsequent analysis. To avoid deviations from the protocol due to variations in the investigators’ explanations, we prepared formulated explanations to possible questions from the participants in advance.

### Outcomes

Study outcomes will be evaluated by obstetricians in charge of antenatal check-ups. At each antenatal check-up, the cephalic version is confirmed by checking the fetal position using ultrasonography.

### Primary outcome

The primary outcome is to evaluate whether serial instructions of the lateral or reverse lateral positions can reduce fetuses in breech presentation in women between 28 + 0 and 30 + 0 weeks of gestation, compared with expectant management care. The reduction of breech fetuses will be determined using the proportion of cephalic fetuses in each group at term (37 weeks). The cephalic fetuses will be confirmed by ultrasonography at 37 weeks visit and is defined as a categorical variable.

### Secondary outcomes

The secondary outcomes are cesarean delivery, cesarean delivery for breech presentation, cephalic presentation 2, 4, and 6 weeks later, and at delivery, recurrent breech presentation after cephalic version, and adverse effects. The cephalic version is recorded as a categorical variable, and values evaluated at each visit or delivery will be used in the analysis. The recurrent breech rate will be compared by the percentage of cumulative occurrences from the time of participation to the end of delivery, and adverse events will be compared by the cumulative number of occurrences from participation to delivery in both groups. For other outcomes, the percentage of cephalic fetuses at each time point will be compared between the two groups. Only the recurrent breech position will be compared between groups converted to a cephalic presentation at least once. All adverse effects and unexpected events will be recorded and discussed among researchers on a case-by-case basis.

### Trial duration

This trial is expected to take approximately 3 years to complete. We reviewed our medical records to calculate the number of people diagnosed with pelvic position each month and calculated the recruitment period based on the frequency with which eligible patients appeared and the assumption of approximately 10% refusal to participate in the study. In addition, the enrollment period was extended for 6 months to allow more time to take into account the number of dropouts. In our previous retrospective cohort study, 70 participants were included in the study in 12 months.

### Data collection and management

Data will be collected at the enrolment, each pregnancy check-up 2 weeks after the instruction until delivery, and after delivery. Table 1 shows the schedule of processes during the study. During enrollment, we will record age, parity, height, weight, presentation, breech type, fetal back position, the position of the placenta, amniotic fluid volume, pregnancy methods, and complications. At each pregnancy check-up, we will record the presentation, breech type, fetal back position, and positions taken at home. If the right and left sides of the fetus are not clearly defined, the classification is made according to whether the fetus is slightly closer to the midline of the abdomen to one side or the other. If the fetal dorsum is judged to be almost in the center, it is judged according to whether the fetal legs are closer to the left or right side of the midline. Postures taken at home are recorded by the subjects themselves, which may result in missing data. The body posture recording form will be collected at every 2-week visit, and if the form cannot be collected, the participants are asked to fill it out on the spot to prevent missing data. Other data are routinely collected at every antenatal checkup and at delivery and therefore are considered to have few missing data. Data collection forms are available upon reasonable request from the corresponding author. After delivery, we will also record gestational age at delivery, mode of delivery, birth weight, Apgar scores at 1 and 5 min, umbilical artery pH at birth, the amount of bleeding during delivery, and complications. No personally identifiable information will be recorded and a research number will be assigned to each participant. Withdrawal from the trial will be recorded and reported. If a participant is transferred to another hospital, there is a possibility of missing outcomes, and this will be handled by inquiring about medical information at the hospital to which the patient is being transferred. All missing values will be reported. All data will be double-checked for accuracy and falsification. Data is password protected and access to the computer is restricted to specific statisticians. The database will be destroyed 5 years after the completion of the research or 3 years after the publication of the results.

**Table 1:** Schedule of processes during the BRLT study

Timepoint	28 to30 weeks	Check-up every 2 weeks	Delivery
Enrollment			
Diagnosis	x		
Eligibility screen	x		
Informed consent	x		
Randomization	x		
Interventions			
Postural instruction for the intervention group	x	x	
Expectant management care for control group	x	x	
Recording of the daily position record form	x	x	x
Confirmation of adverse events		x	x
Assessment			
Characteristics of participants	x		
Complications	x	x	x
Deviation from the protocol	x	x	x
Ultrasonography			
Presentation	x	x	x
Position of the fetal back	x	x	x
Breech type	x	x	x
Position of the placenta	x		
Delivery			
Mode of delivery			x
Findings at birth			x

### Statistical analysis

An intention-to-treat and per-protocol analysis will be performed. Of those, intention-to-treat analysis will be reported as the main result.

Primary outcome and secondary outcomes were two-category data, so the comparison between two groups will be conducted by *χ*^2^ test (Pearson’s or Fisher’s test). Unadjusted risk ratios will be calculated as the main result with a 95% confidence interval. The absolute risk reduction will also be calculated. The student’s *t*-test will be conducted when continuous variables are normally distributed, and the Mann–Whitney *U* test will be conducted when they are not normally distributed. If there is a bias in the characteristics between the two groups, an adjusted analysis will also be conducted using that factor as a confounding factor. Subgroup analysis will also be performed separately for primiparous and multiparous women.

The intention-to-treat analysis, which will be treated as the main outcome, will include protocol deviations and will compare all participants for whom results are available between the two groups. A patient is considered to have successfully performed the lateral position if she has performed it on the correct side for at least 45 min in at least half of the 2-week period. Whether or not the patient was able to perform the postural therapy as per protocol is recorded in a categorical variable. The per-protocol analysis will compare the cases in the intervention group with a success rate of > 50% in the lateral position with the control group as a whole and compare the cases in the intervention group with a success rate of > 50% in the lateral position with the control group with a rate of correct lateral positioning < 50%.

For missing values, we will report the number of missing values and use the mean imputation for continuous variables and the pairwise deletion for categorical variables.

A *p*-value < 0.05 will be considered statistically significant, and a two-sided test will be performed in all analyses. Statistical analyses will be conducted using the Statistical Package for the Social Sciences software for Windows (version 26.0, IBM Corp., Armonk, NY, USA). Other details of the statistical analysis plan are available upon reasonable request from the corresponding author.

### Interim analysis

When the outcomes of 100 patients have been confirmed, an interim analysis will be performed only once. The objective is to determine whether there is an evident disadvantage to the participants in continuing the study, such as a significant difference in the cesarean section rate. In the interim analysis, a *p*-value < 0.01 will be considered statistically significant. Other analysis methods will be equivalent to the final analysis.

### Adverse events

According to a meta-analysis, sleeping on the left or right sides appears equally safe [20]. Although a review of the literature suggests that the interventions in this study will have no adverse effects, any health damage induced by this research will be treated within the scope of health insurance.

### Early termination of the trial

The ethics committee did not require a data monitoring committee because it is not a trial with severe outcomes such as death, it is not a huge, long-term trial, and the intervention is not invasive and is considered safe in the literature and theory. However, to address obvious disadvantages of participants, such as differences in cesarean section rates, the statistician would perform an interim analysis once the outcomes of 100 patients, half of the planned number of patients, have been confirmed. The principal investigator and research assistants will periodically check for any deviations from the protocol or missing data and discuss whether the protocol needs to be amended if necessary. The hospital director plays the roles of overseeing study conduct, data quality, and patient safety. The status of the study implementation will be reported to the hospital director on a regular basis (at least once a year). If unanticipated severe adverse events or clear disadvantages to research participants are observed during the trial, the hospital director will be notified immediately, and the investigators and those involved will discuss early termination.

### Ethics

This study is in accordance with the Declaration of Helsinki (64th World Medical Association General Assembly, Fortaleza, Brazil, October 2013), and it complies with Japanese Ethical Guidelines for Medical and Health Research Involving Human Subjects (Public Notice of the Ministry of Education, Culture, Sports, Science and Technology, the Ministry of Health, Labour and Welfare, and the Ministry of Economy, Trade and Industry, Tokyo, Japan, March 2015). Furthermore, this protocol was designed in line with SPIRIT (Standard Protocol Items: Recommendations for Interventional Trials) 2013 [29]. The Ethics Committee of Nippon Medical School Musashikosugi Hospital approved this protocol on 9 March 2021 (ID: 599–2-69). The study was registered in UMIN Clinical Trials Registry (ID: UMIN000043613) on 15 March 2021, https://center6.umin.ac.jp/cgi-open-bin/ctr_e/ctr_view.cgi?recptno=R000049800. Table 2 shows the WHO trial registry data set. This paper is based on protocol version 2.0 (8 June 2022). Important protocol modifications will be reviewed by the Ethics Committee on a case-by-case basis and communicated to the relevant parties.

**Table 2:** Trial registration data

**Data category**	**Information**
Primary registry and trial identifying number	UMIN000043613
Date of registration in primary registry	15 March 2021
Secondary identifying numbers	R000049800, 599–2-64
Source of monetary or material support	None, self-funding
Sponsor	None
Contact for public queries	Hiroki Shinmura MD, h-shimmura@nms.ac.jp
Contact for scientific queries	Hiroki Shinmura MD, Department of Obstetrics and Gynecology, Kawasaki, Japan
Public title	A randomized controlled trial of cephalic version for breech presentation in the third trimester by lateral postural management without knee-chest position (BRLT)
Scientific title	A randomized controlled trial of cephalic version for breech presentation in the third trimester by lateral postural management without knee–chest position
Countries of recruitment	Japan
Health condition or problem studied	Breech presentation
Interventions	Interventions: Women of the interventional group are going to be instructed to lie on their right side several times a day when the fetal back was left side, or to lie on their left side when the fetal back was right side. After cephalic version, they are going to be instructed the other side. Other genupectoral management is not recommended
	Control: Women of the control group are going to receive expectant management care complying with Guidelines for obstetrical practice in Japan 2020
Key inclusion and exclusion criteria	Inclusion criteria: Women who are diagnosed with breech presentation by ultrasonography between 28 weeks 0 days and 30 weeks 0 days of gestation at a single institution were eligible for inclusion
	Exclusion criteria: The exclusion criteria were scheduled cesarean delivery (included placenta previa, history of cesarean delivery, and history of myomectomy), multiple pregnancy, transverse position, scheduled delivery at another hospital, other genupectoral management, and complications such as heart disease
Study type	Interventional
	Allocation: randomized; intervention model: parallel assignment; masking: open—no one is blinded
	Primary purpose:
	Phase III
Date of first enrolment	April 2021
Target sample size	200
Recruitment status	Recruiting
Primary outcome	The percentage of fetuses in cephalic presentation at term
Secondary outcomes	The percentage of cephalic presentation 2 weeks later, 4 weeks later, 6 weeks later, and at delivery, the rate of cesarean delivery, the rate of cesarean delivery for breech presentation, recurrent breech presentation after cephalic version, and adverse effects

### Auditing

The ethics committee did not require auditing for this trial. Annually, the progress of the study will be reported to the hospital director. If an ethical problem occurs, it will be immediately reported to the hospital director, and discussions among researchers and the people concerned should be held promptly, including early termination of the trial.

### Dissemination

The result of this trial will be presented at conferences and published in peer-reviewed journals. When publishing a paper, we do not use professional writers, and the first author will be the principal investigator.

### Patient and public involvement

Patients and the public were not involved in the design, the conduct, or the dissemination of this trial.

## Discussion

To the best of our knowledge, this study will be the largest-scale randomized controlled trial to evaluate the effect of postural management for breech presentation. There are a certain number of cases in which the external cephalic version is unsuccessful [30], and there is no established treatment for breech presentation before 36 weeks. Thus, if the result is positive, it may be a valid indicator of the treatment of breech presentation before 36 weeks.

Furthermore, this is the first study to validate the effectiveness of the reverse lateral position. It is a modification of the lateral position theory, which was practiced originally by the principal author. It is thought to stabilize the fetal position by lying on the same side as the fetal back using the effect of gravity. The literature indicates that, as with the lateral position, no adverse events are likely to occur [20]. If this study proves the usefulness of the reverse lateral position, it also could be practiced as maintenance therapy after the external cephalic version.

Although this study is open-labelled, it is difficult to blind studies on postural therapy, and all previous postural studies deemed to be of high quality are also open-labelled [12, 31–36]. Therefore, outcome measures were set to be independent of observer bias. Furthermore, predetermined examples of explanations were prepared to reduce the bias caused by the investigator’s explanations.

To emphasize clinical applicability, we will not prohibit the side-lying method in the control group. Therefore, women in the control group may have taken the lateral position accidentally or intentionally without being instructed. To evaluate this impact, we would ask all participants to record what position they are in when lying down.

In summary, the BRLT study, for which we described the protocol here, will evaluate the effectiveness of the lateral position management for breech presentation in the third trimester of pregnancy. If the lateral positioning technique is proven to be effective in treating breech presentation, it may provide a very simple, less painful, and safe option for treating breech presentation before 36 weeks, and it may impact breech presentation treatment.

## Trial status

The current version of the protocol is 2.0, dated 8 June 2022. Recruitment for this trial has already begun on 1 April 2021. The expected completion date of the study is March 2024.

## Supplementary Information

**Additional file 1.** SPIRIT Checklist for Trials.

## Data Availability

The data that support this study are available from the corresponding author upon reasonable request.
